# Complete chloroplast genome of *Camellia chekiangoleosa* (Theaceae), a shrub with gorgeous flowers and rich seed oil

**DOI:** 10.1080/23802359.2021.1884026

**Published:** 2021-03-15

**Authors:** Xin Yin, Tian Li, Bin Huang, Lian Xu, Qiang Wen

**Affiliations:** a Co-Innovation Center for Sustainable Forestry in Southern China, Nanjing Forestry University, Nanjing, China; b Jiangxi Provincial Key Laboratory of Camellia Germplasm Conservation and Utilization, Jiangxi Academy of Forestry, Nanchang, China

**Keywords:** *Camellia chekiangoleosa*, chloroplast genome, phylogenetic analysis

## Abstract

*Camellia chekiangoleosa* Hu is an oil-tea *Camellia* species with high economic and nutritional value in the south of China. In this study, the chloroplast genome of *C. chekiangoleosa* was determined by Illumina Miseq platform. The whole chloroplast genome is 156,971 bp in length, containing a large single-copy region (LSC, 86,673 bp), a small single-copy region (SSC, 18,394 bp), and a pair of inverted repeat regions (IRa and IRb, 25,952 bp). There is a total of 113 genes in the complete chloroplast genome of which 19 genes are repeated in the IR regions. In addition, the phylogenetic tree revealed a close relationship between *C. chekiangoleosa* and *C. japonica*. The complete chloroplast genome will contribute to further studies on phylogeny and conservation of *C. chekiangoleosa* and related taxa in *Camellia* of Theaceae.

*Camellia chekiangoleosa* is an important species of genus *Camellia* (*Theaceae*), provides edible camellia oil, which is rich in unsaturated fatty acid, camellia glycosides, camellia saponins, tea polyphenols, and many other active substances beneficial to health (Yong 2013; Yu et al. 2017). It is mainly distributed in Jiangxi, Zhejiang, Hunan and Fujian Province, south of China. The oil production of *C. chekiangoleosa* is not as much as *C. oleifera,* but it has a higher ratio of nutrients. In addition, *C. chekiangoleosa* has ornamental value due to its red and white flowers.

The fresh and healthy leaves of *C. chekiangoleosa* were sampled from Nanchang (28°44′21.26ʺN, 115°49′5.42ʺE), and were stored in the Key Laboratory of *Camellia* Germplasm Conservation and Utilization, Jiangxi Academy of Forestry (voucher specimen: TB004).Chloroplast DNA was isolated from the fresh leaves using Chloroplast DNA Isolation Kit (Sigma-Aldrich, USA) before being sequenced entirely using Next-generation sequencing (Illumina Miseq platform). The sequence data were automatically annotated by the web-program DOGMA (http://dogma.ccbb.utexas.edu/) and checked with the chloroplast genome of *C. crapnelliana* as a reference (Wyman et al. 2004). The chloroplast genome of *C. chekiangoleosa* was uploaded to GenBank (Accession Number: MG431968).

The whole chloroplast genome was 156,946 bp in length with 37.30% GC content and contained a pair of IR regions (25,954 bp) separated by LSC (86,656 bp) and SSC (18,382 bp) regions. The chloroplast genome contained 113 genes, including 80 protein-coding genes, 29 tRNA genes, and four rRNA genes. There were eight protein-coding genes, four rRNA genes, and seven tRNA genes repeated in the IR region among them.

In order to validate the phylogenetic relationship, the whole chloroplast genome sequence of *C. chekiangoleosa* and18 related taxa were aligned using MAFFT v7.271 (Katoh and Standley 2013). Then the alignment will be used in the Maximum Likelihood (ML) analysis for phylogeny, which performed by RaxML with the SH-aLRT test and 1000 bootstraps, with 2 species from *Symplocos* as an outgroup (Silvestro and Michalak 2012). Finally, the tree was visualized and beautified using Figtree v1.4.3. As shown in Figure 1, *Camellia* species formed a monophyletic clade with 100% bootstrap support value, while *C. chekiangoleosa* and *C. japonica*were sister relationship.

**Figure 1.**
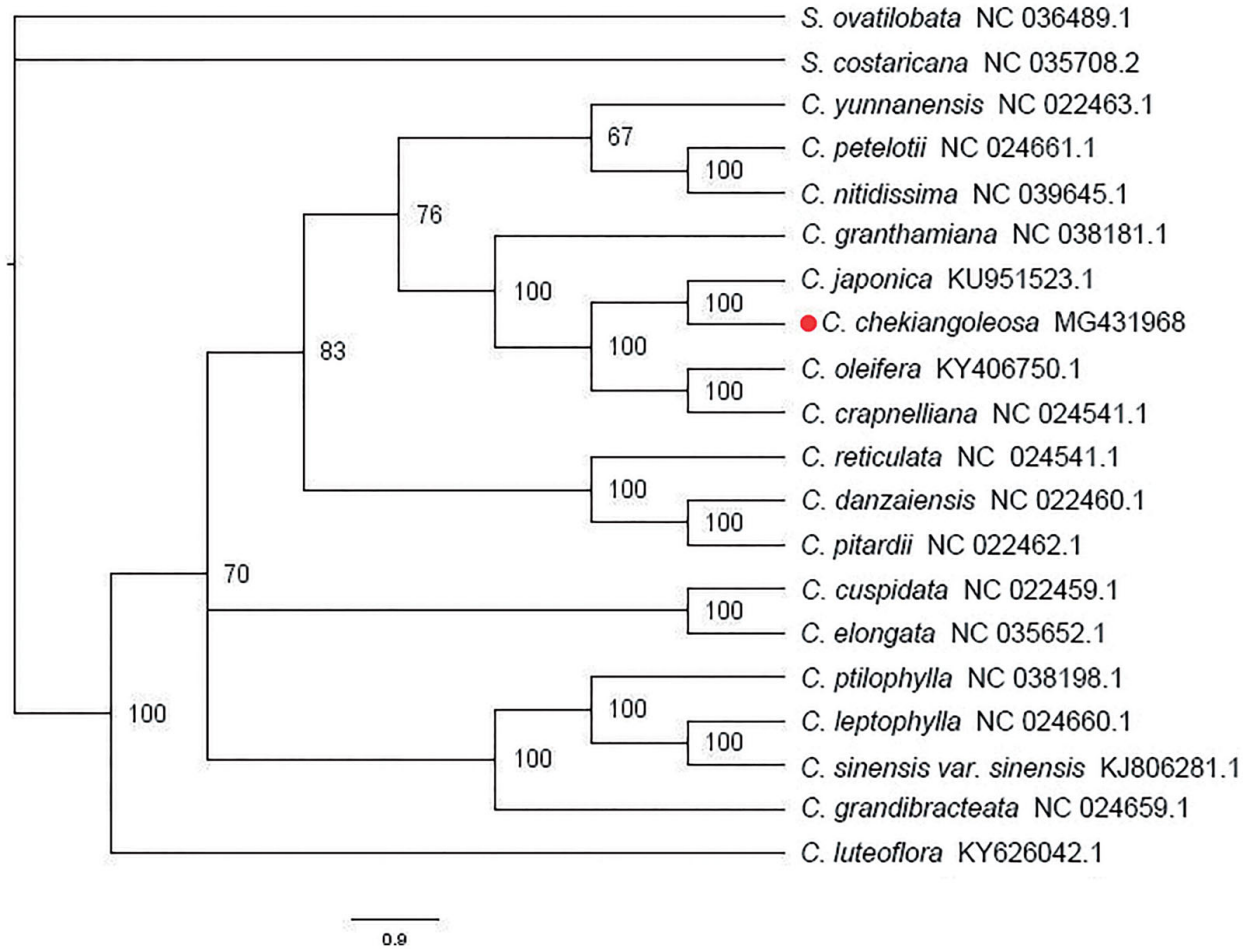
A phylogenetic tree constructed based on 18 complete chloroplast genomes of *Camellia* species with 2 of *Symplocos* as an outgroup, while the number on the right of nodes showed the bootstrap value.

## Data Availability

Chloroplast data supporting this study are openly available in the National Center for Biotechnology Information (https://www.ncbi.nlm.nih.gov/), reference number (MG431968).

## References

[CIT0001] Katoh K, Standley DM. 2013. MAFFT multiple sequence alignment software version 7: improvements in performance and usability. Mol Biol Evol. 30(4):772–780. 23329690 10.1093/molbev/mst010 PMC3603318

[CIT0003] Silvestro D, Michalak I. 2012. raxmlGUI: a graphical front-end for RAxML. Org Divers Evol. 12(4):335–337.

[CIT0005] Wyman SK, Jansen RK, Boore JL. 2004. Automatic annotation of organellar genomes with DOGMA. Bioinformatics. 20(17):3252–3255. 15180927 10.1093/bioinformatics/bth352

[CIT0006] Yong H. 2013. Analysis of *Camellia chekiangoleosa* genetic diversity based on SRAP markers. Scientia Silvae Sinicae. 49(3):43–50.

[CIT0007] Yu XQ, Gao LM, Soltis DE, Soltis PS, Yang JB, Fang L, Yang SX, Li DZ. 2017. Insights into the historical assembly of East Asian subtropical evergreen broadleaved forests revealed by the temporal history of the tea family. New Phytol. 215(3):1235–1248. 28695680 10.1111/nph.14683

